# The identity of the Neotropical stingless bee *Frieseomelitta meadewaldoi* (Cockerell, 1915) (Hymenoptera, Apidae)

**DOI:** 10.3897/zookeys.111.1345

**Published:** 2011-06-22

**Authors:** Favízia Freitas de Oliveira, Danúncia Urban, Michael S. Engel

**Affiliations:** 1 Laboratório de Bionomia, Biogeografia e Sistemática de Insetos (BIOSIS), Departamento de Zoologia, Instituto de Biologia, Universidade Federal da Bahia, Rua Barão de Jeremoabo, s/n, Campus Universitário de Ondina, Salvador, CEP 40170-115, Bahia, Brazil; 2 Departamento de Zoologia, Universidade Federal do Paraná, Centro Politécnico, Setor de Ciências Biológicas, Caixa Postal 19020, Curitiba, CEP 81531-990, Paraná, Brazil; 3 Division of Entomology, Natural History Museum, and Department of Ecology & Evolutionary Biology, 1501 Crestline Drive – Suite 140, University of Kansas, Lawrence, Kansas 66049-2811, USA

**Keywords:** Apoidea, Anthophila, Apidae, Apinae, Meliponini, *Trigona*, *Frieseomelitta*, taxonomy

## Abstract

A new study of a surviving syntype of *Trigona meadewaldoi* Cockerell, 1915, was undertaken and several widely employed names for Neotropical stingless bees recognized as junior synonyms. A lectotype is designated for *Trigona meadewaldoi* and the following new synonymies established: *Tetragona francoi* Moure, 1946, and *Trigona (Frieseomelitta) freiremaiai* Moure, 1963. These nomenclatural matters are here settled and the species thoroughly characterized in advance of a forthcoming phylogenetic consideration of the genus *Frieseomelitta* von Ihering, 1912.

## Introduction

*Frieseomelitta* von Ihering, 1912 is a genus of New World stingless bees (Apinae, Meliponini), with a wide geographic range occurring from southwestern Mexico (Sinaloa) to the southeast of Brazil (São Paulo), and can be found in forests, cerrado, caatinga, and mountainous regions, reaching an elevation of about 1600 m (Iguala, Guerrero, México). Species are moderately aggressive and nest in cavities in living or dead trees (dry), fence posts, &c. In general they apparently are not well adapted to anthropogenic environments, with few records originating in urban areas, with the exception of *Frieseomelitta trichocerata* Moure, 1988 which is quite common in Manaus, Brazil. Species of *Frieseomelitta* are popularly known as little black girls, lacemakers, zamboque, pitch, abreu, long legs, black marmalade, white marmalade, white wings, white girls, or ties nêgo.

Nineteen species are presently recognized, although no formal revision has yet been published, and at least seven undescribed species are known (Oliveira et al. in prep.). The hitherto recognized species are *Frieseomelitta flavicornis* (Fabricius, 1798), *Frieseomelitta varia* (Lepeletier de Saint Fargeau, 1836); *Frieseomelitta longipes* (Smith, 1854); *Frieseomelitta nigra* (Cresson, 1878); *Frieseomelitta paupera* (Provancher, 1888); *Frieseomelitta portoi* (Friese, 1900); *Frieseomelitta doederleini* (Friese, 1900); *Frieseomelitta lehmanni* (Friese, 1901); *Frieseomelitta silvestrii* (Friese, 1902); *Frieseomelitta meadewaldoi* (Cockerell, 1915); *Frieseomelitta parastigma* (Cockerell, 1918); *Frieseomelitta pura* (Cockerell, 1920); *Frieseomelitta paranigra* (Schwarz, 1940); *Frieseomelitta francoi* (Moure, 1946); *Frieseomelitta dispar* (Moure, 1950); *Frieseomelitta freiremaiai* (Moure, 1963); *Frieseomelitta savannensis* (Roubik, 1980); *Frieseomelitta trichocerata*, and *Frieseomelitta languida* Moure, 1989. Herein we attempt to clarify the taxonomic status of three of these names in advance of a phylogenetic study of the genus (Oliveira et al. in prep.).

Smith (1854) established the species, *Trigona dorsalis* (today *Tetragona dorsalis*), for a stingless bee from “Brazil (Pará)”. Later (Smith, 1863) redescribed the species based on a series of 10 individuals presented at the International Exhibition of 1862. On pages 499–500 of his work Smith (1863) states, “For the purpose of examination, when describing the species in the present paper, I was supplied with ten examples of each...” and so it is presumed (taking his statement at face value) that there was originally a series upon which he based his description of *Tetragona dorsalis* in 1863.

More than a half century later, Cockerell (1915) noted the incongruence of the two descriptions published by Smith under the name *Tetragona dorsalis*, and after examining the 1854 type for the species, concluded that the 1854 and 1863 descriptions referred to different species altogether. Cockerell (op. cit.) therefore considered the specimen redescribed as *Tetragona dorsalis* by Smith (1863) to be a separate species from the one Smith described under this name in 1854. Accordingly, Cockerell (1915) proposed the name *Trigona meadewaldoi* as a replacement for the species described by Smith in 1863, selecting as epithet a patronym for Geoffroy Meade-Waldo (1884–1916). However, Cockerell never did find the specimens described by Smith (1863).

From 1915 until recently, most melittologists had interpreted *Trigona meadewaldoi* to be a junior synonym of *Trigona doederleini* Friese, 1900, principally by the fact that many individuals of the latter species were found among specimens from Smith’s collection. Given that the 1863 type series was apparently missing or unrecognized, there was no basis upon which to contradict this anecdotal conclusion. While the 1863 description of *Tetragona dorsalis* could be applied in part to *Trigona doederleini*, there are sufficient discrepancies to suspect that the specimens described by Smith in 1863 were not necessarily conspecific with *Trigona doederleini*. One of the significant differences between Smith’s 1854 *Tetragona dorsalis* (as noted, now a valid species of *Tetragona*) and his “*Tetragona dorsalis*” of 1863 (clearly a species of *Frieseomelitta*) is the coloration of the face. For example, Smith (1854) notes “Head black; the clypeus, a triangular spot above, the face on each side, the scape in front, and the mandibles, yellow” and “wings testaceous” (1854: 411), while in 1863 he states, “Pale ferruginous, with the head and thorax black above; clypeus, mandibles and antennae pale ferruginous; also a narrow pale line at the inner orbits of the eyes” and “alis hyalinis” (1863: 510). The latter account aptly describes the face and wings of two other potentially conspecific species, *Tetragona francoi* Moure, 1946 and *Trigona freiremaiai* Moure, 1963 (e.g., Figs 4, 5, 7, 8, 10) (*vide infra*). All of these specimens also have identical metatibiae which are taciform in shape (e.g., Figs 3, 5, 10, 14), rather than the more bulky form of *Trigona doederleini* and other species (e.g., Fig. 13). Moreover, these same features can serve to distinguish *Trigona doederleini* from *Trigona meadewaldoi* (a.k.a., the “*Tetragona dorsalis*” of 1863). Indeed, a study of Smith’s collection of material labeled by Herbert F. Schwarz as “*Trigona meadewaldoi* = *Trigona doederleini*” and those bearing the label “18” mentioned by Cockerell (1915) are identical with *Trigona doederleini* (type material of *Trigona doederleini* was studied by Oliveira 2003 and the lectotype was re-examined for the purposes of the present study, *vide infra*) but do not match Smith’s 1863 description of “*Tetragona dorsalis*” (a.k.a., *Trigona meadewaldoi*). This led us to believe that the material examined by previous authors was not of Smith’s syntype series. Baker (1993) noted that Smith’s series from the 1863 paper was originally broken up between London and Oxford, and that,

“The second set, in another part of the type collection (UMO 2), apparently, unfortunately, not seen by Schwarz [Herbert F. Schwarz], is grouped with various vespoids in a tray with the note: ‘Honey bees and Wasps of South America. Exhibited in the International Exhibition of 1862. Presented by John Miers Esq F.R.S. 1865. See Memoir by F. Smith in Trans. Entom. Society’”

(Baker 1993: 232).

As noted by Baker (op. cit.) this set comprises two specimens of each species examined by Smith (1863). For material of “*Tetragona dorsalis*” there are two specimens, one bearing a blue label with “*Trigona dorsalis* Sm” in Smith’s hand, the other nothing more than a label reading “18” (as was material Cockerell had available to him). Both of these specimens are of *Trigona doederleini* and do not match Smith’s (1863) description. It can only be presumed that Smith’s original series was mixed and that he based his description on a subset of these specimens.

**Figures1–7.**
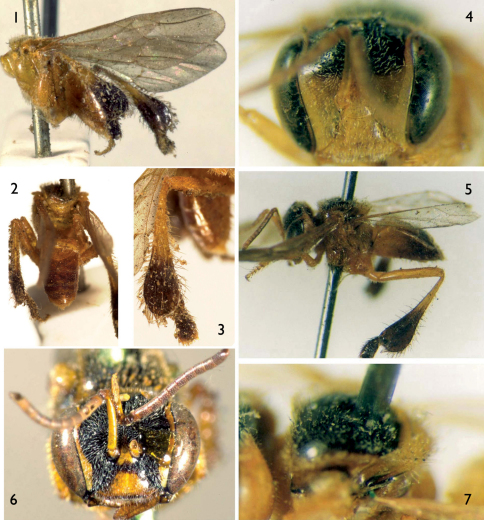
*Trigona meadewaldoi* Cockerell, 1915 (lectotype, NHML), *Tetragona francoi* Moure, 1946 (holotype, DZUP), and *Trigona doederleini* Friese, 1900 (lectotype, AMNH) (all are workers). **1** Lateral habitus of *Trigona meadewaldoi* lectotype as preserved **2** Dorsal view of metasoma of *Trigona meadewaldoi* lectotype **3** External surface of metatibia of *Trigona meadewaldoi* lectotype **4** Facial view of *Tetragona francoi* holotype **5** Lateral habitus of *Tetragona francoi* holotype **6** Facial view of *Trigona doederleini* lectotype **7** Dorsal oblique view of mesoscutum and mesoscutellum of *Tetragona francoi* holotype.

Among material from Smith’s collection in The Natural History Museum, London, a single worker individual was discovered missing its head and bearing a blue labeled signed by Smith as “*Trigona dorsalis*” (Figs 1–3, 14). This specimen was apparently not examined by previous authors when studying Neotropical Meliponini (e.g., Cockerell, Schwarz, Moure, or Camargo as none of these authors had placed their usual identification labels with the specimen). In preserved details, this specimen matches perfectly Smith’s (1863) description (obviously those characters of the head cannot be confirmed as the head was lost at some point in the past). It was therefore suspected that this could be one of the individuals upon which Smith had based his 1863 redescription. Given that this specimen is almost certainly from Smith’s 1863 exhibition series and that, unlike other specimens apparently from that series, it closely matches his description, we conclude that this is material from which he based his account. No other potential syntypes are known in collections and we accordingly select herein this individual to serve as the lectotype for *Trigona meadewaldoi*.

As alluded to earlier, two further epithets come into play. *Tetragona francoi* was described in detail by Moure (1946), based on a single worker from Riachuelo (Sergipe, Brazil) and collected by the famed agronomist Dr. A. Franco Filho, to whom Moure dedicated the species. Later, Moure (1963) described *Trigona* (*Frieseomelitta freiremaiai*, noting however that the material could prove to be conspecific with, and thereby a junior synonym of, *Tetragona francoi*. Indeed, having now examined the type material for both *Tetragona francoi* and *Trigona freiremaiai* it is clear that the minor differences mentioned by Moure (1963) are geographic variations of one species. In addition, both are proposed as junior synonyms of *Trigona meadewaldoi*, the identity of which is established by the newly recognized holotype (*supra*), and which takes priority over both of Moure’s epithets. Smith’s (1863) account indicating “head and thorax black above” (p. 510) agrees perfectly with both of these putative species in which the head is yellow (or pale yellow to orange-yellow or testaceous in some older specimens) except for a black rectangle on the upper part of the face and vertex (above the upper tangent of the antennal alveoli to the vertex, extending posterior to the ocelli until the occiput), as well as the mesosoma which has a dark brown to black mesoscutum. Although Smith (1863) referred to the wings as hyaline, the wing membrane tends to be very faintly infumate. In *Trigona doederleini* the mesosoma is similarly yellow with a black mesoscutum but in this species the head is entirely black with yellow markings on the clypeus, supraclypeal area, paraocular area, and gena. Oliveira (2003) clarified the taxonomic status of *Trigona doederleini*.

## Material and methods

The type material considered herein is deposited in the following institutions: AMNH, Division of Invertebrate Zoology (Entomology), American Museum of Natural History, New York, USA; DZUP, Coleção de Entomologia Pe. J.S. Moure Departamento de Zoologia da Universidade Federal do Paraná, Curitiba, Brazil; and NHML, Department of Entomology, The Natural History Museum, London, United Kingdom.

Given the incomplete nature of some material we based the metrics on the most complete of the type material, basically that of the holotype of *Tetragona francoi*) and were made using an ocular micrometer (with precision of 0.001 mm) on a Leica MZ12.5 stereomicroscope. All measurements are in millimeters. Measurements used herein are as follows: length of forewing measured from apex of costal sclerite to apex wing; diameters or width of structures were obtained by taking the maximum diameter or width (e.g., head width, clypeal width, compound eye width, scape diameter, meso- and metafemoral width, meso- and metatibial width, meso- and metabasitarsal width); total body length refers to distance from apex of clypeus to posterior margin of apicalmost metasomal segment; length of head in frontal view was taken from crest of vertex to medioapical margin of clypeus; width of head in frontal view corresponds to maximum width including compound eyes; height of compound eye in lateral view was taken as its maximum height (length); ocellorbital distance refers to the shortest distance in laterodorsal view between lateral ocellus and upper inner margin of compound eye; interocellar distance is that between the lateral and median ocelli; upper interorbital refers to the distance between the inner margins of the upper compound eye orbits; middle interorbital refers to the distance between compound eyes along a line approximately one alveolar diameter above the upper alveolar tangent; lower interorbital refers to the distance between the inner margins of the lower compound eye orbits; antennal scape length was measured from its apex to its base excluding the radical; lengths of femora, tibiae, and basitarsi were taken along their longitudinal axis from apex to joint with preceding podite.

In addition to morphometric measurements, we examined a suite of morphological characters commonly used in meliponine systematics, including, but not limited to, pilosity, body coloration, and shape of the head, legs, and mesosoma. Morphological terminology follows that of Urban (1967), Camargo et al. (1967), Engel (2001), and Michener (2007), with the addition of the term “taciformes” (for metatibial shape) referring to that form resembling a baseball bat. The following abbreviations are used: DA, alveolus diameter; DE, scape diameter; DP, puncture diameter; T, metasomal tergum. All characters included in the “Diagnosis” refer to workers as this is the caste most frequently found in Nature, while queens and drones are often unknown. In addition, the characters provided are those that make it most easy to recognize the species and not all are necessarily autapomorphic (Oliveira et al. in prep.).

## Genus Frieseomelitta von Ihering, 1912

### Frieseomelitta meadewaldoi

(Cockerell, 1915)

http://species-id.net/wiki/Frieseomelitta_meadewaldoi

Figs 1–5, 7 –12, 14

Trigona dorsalis Smith; Smith 1863: 504, 510 [misidentification, *non Trigona dorsalis* Smith, 1854].
Trigona meadewaldoi Cockerell 1915: 32. *Nomen novum pro Trigona dorsalis* Smith, 1863 *non Trigona dorsalis* Smith, 1854.
Tetragona francoi Moure 1946: 437–438. Moure 1963: 39. Syn. n.
Trigona (Tetragona) francoi  (Moure); Moure 1951: 44.
Trigona(Frieseomelitta) francoi  (Moure); Wille 1962: 179.
Trigona(Frieseomelitta) meadewaldoi  Cockerell; Wille 1962: 179.
Trigona(Frieseomelitta) freiremaiai Moure 1963: 39-43. Wille 1962: 179; Cruz-Landim 1963: 2–4 (as *Friseomelitta* [sic]); Sakagami et al. 1963: 116, 119–121, 124, 126–128; Kerr and Esch 1965: 532, 536; Kerr et al. 1967: 279, 282; Cruz-Landim 1967: 194, 254, 266, 267, 270; Akahira and Beig 1967: 166, 169, 172, 173, 180–181, 184 (Figs 15, 16); Michener 1990: 103. Syn. n.
Frieseomelitta freiremaiai (Moure); Nogueira-Neto 1963: 115; Abdalla 2002: 135.
Trigona(Tetragona) freiremaiai  (Moure); Wille and Michener 1973, pp. 14, 24, 48, 59, 70.
Trigona freiremaiai (Moure); Costa 2002: 94.
Frieseomelitta francoi (Moure); Silveira et al. 2002: 87.
Frieseomelitta doederleini (Friese, 1900); Camargo and Pedro 2007: 291 [misidentification].

#### Lectotype (here designated).

Worker (NHML, Figs 1–3, 14): labeled “*Trigona dorsalis* Sm” in Smith’s hand on a blue label. Locality given solely as “Brazil” by Smith (1863; *vide etiam* Comments, *infra*) in his redescription of *Trigona dorsalis* Smith, 1854 (in 1854 he provided “Brasil (Pará)” as the type locality for *Tetragona dorsalis*).

#### Lectotype (here designated).

**Figures 8–14.**
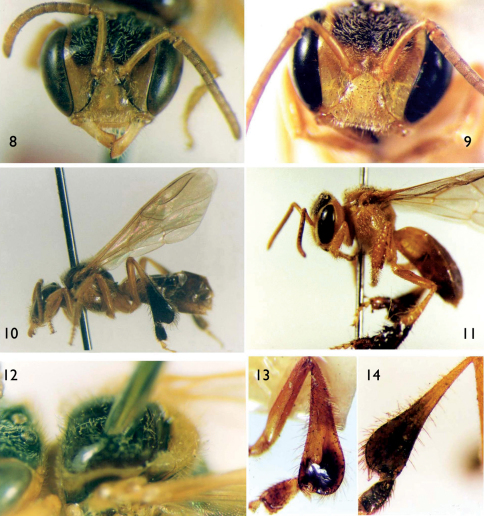
*Trigona meadewaldoi* Cockerell, 1915 (lectotype, NHML; non-type, DZUP), and *Trigona doederleini* Friese, 1900 (lectotype, AMNH) (all are workers). **8** Facial view of *Trigona freiremaiai* holotype. **9** Facial view of *Frieseomelitta meadewaldoi* from the State of Bahia, Brazil (non-type material). **10** Lateral habitus of *Trigona freiremaiai* holotype. **11** Lateral habitus of *Frieseomelitta meadewaldoi* (non-type material). **12** Dorsal oblique view of mesoscutum and mesoscutellum of *Trigona freiremaiai* holotype. **13** Metatibia of *Trigona doederleini* lectotype. **14** Metatibia of *Trigona meadewaldoi* lectotype.

Worker holotype (DZUP, Figs 4, 5, 7) of *Tetragona francoi* Moure, 1946; labeled “Riachuelo, Sergipe, Brasil, R. Franco col.”. Holotype worker (DZUP, Figs 8, 10, 12) of *Trigona* (*Frieseomelitta*) *freiremaiai* Moure, 1963; labeled “Guarapari, ES, Brasil, II.1961”; and 21 paratypes, workers of the same species, labeled “Guarapari, Espírito Santo, Brasil: II.1961” [n=7], “IX.1960, M. Alvarenga col.” [n=2]; “Maracás, Bahia, Brasil: 970m, VI.1961, F.M. Oliveira col.” [n=5], and “VI.1961” [n=7]. Lectotype worker (AMNH 25290, Figs 6, 13) of *Trigona doederleini* Friese, 1900; labeled “Chiriqui, *Trigona doederleini* Friese, 1910” and with a typical orange Friese “Typus” label.

#### Diagnosis.

*Worker*: Integument predominantly pale yellow to amber-yellow except dark brown to black on frons (rectangular area), dark brown to black on mesoscutum (margined by yellow lines), dark brown to black on apical two-thirds of metatibia and metabasitarsus; metasoma largely brown except first tergum, basal half of second tergum, and entirety of apicalmost tergum yellow to amber-yellow. Wing membrane faintly infumate, darker on marginal cell and with apical 6% somewhat white. Plumose setae of dorsal surface of mesotibia with long rachis and setal branches restricted to apical one-third of rachis; plumose setae of dorsal surface of mesobasitarsus forming a broad band. Metasoma elongate; metatibia taciform, with inflated aspect (Fig. 14); forewing marginal cell scarcely open at apex; typically six hamuli on leading edge of hind wing.

#### Descriptive notes.

##### Coloration:

Head pale yellow to amber-yellow except for dark brown to black transverse rectangle on upper face extending from above upper alveolar tangent (at a distance of approximately 1 DA) to occiput, bounded laterally by paraocular yellow lines, such paraocular marks even evident on lower yellow portion of face as paler yellow markings, slightly wider below, with greatest width close to tentorial foveae (1.6 DE); genal and paraocular yellow marks join at upper border of compound eye, thereby entirely surrounding orbits; genal marks rather narrow, almost imperceptible, more clearly defined along upper border of compound eye; gena pale yellow to testaceous; clypeus, supraclypeal area, and paraocular area pale yellow; epistomal sulcus brown to dark brown; scape pale amber-yellow to testaceous, with a brownish spot dorsoapically occupying one-third apical length; pedicel and first flagellomere yellowish ventrally; mandibles yellow to amber-yellow, with brown apex; labrum yellow to amber-yellow. Mesosoma yellow to amber-yellow or testaceous except mesoscutum dark brown to black and bordered laterally by large yellow to amber-yellow streaks, such lines a little wider at corners before forming shape of an inverted “J”; tegula yellowish translucent. Wing membranes lightly infumate, darker in marginal cell, apex whitish (apical 6%); veination amber-yellow except R and Rs bordering marginal cell light brown to brown. Legs yellow to amber-yellow or testaceous except dark brown to black on apical two-thirds of metatibia and entirety of metabasitarsus, remaining tarsomeres yellow to amber-yellow. Metasoma largely reddish brown to dark brown; first tergum and basal half of second tergum yellow to testaceous; remaining terga dusky, with apical tergum yellow to testaceous.

##### Pubescence:

Pubescence pale yellow, relatively thin and short. Face with short plumose setae (longest approximately 0.5 DE), such setae with minute rachis and compactly plumose, branches long, such setae semi-decumbent on lower face and semi-erect on frons and vertex (more distinctly evident in this area); thin erect, long, feathery setae intermingled (2 DE), those in paraocular area slightly shorter, those posterior to ocelli longer and more curved; thin, long (2 DE), erect setae between plumose setae, shorter medially in paraocular area and longer and curved posterior to ocelli; setae of scape short and sparse, the longest approximately 0.5 DE, denser along inner margin near base; pubescence of gena simple, very thin, short, and decumbent by comparison with that of face and body, erect setae posterior to ocelli somewhat more dense and with relatively long rachis (about half length) and sparse apical branches. Simpler setae of mesoscutum slightly longer than twice length of plumose setae (2.5 DE), plumose setae with relatively long rachis and poorly branched apically, slightly shorter on disc (1 DE); anterior and lateral borders with setae with shorter rachis and more abundantly branched; setae of mesoscutellum longer (2 and 3 DE for simple and plumose setae, respectively), with long rachis and relatively few branches; mesepisternum with plumose setae and simpler setae relatively thin and long (1.0–2.5 DE and 3 DE, respectively), setae with short rachis (about half length) and relatively sparse apical branching, some with a longer apical filament. Legs with pubescence yellow to pale yellow except corbicular setae, those on internal surface of metatibia dark brown, on inner surface of metabasitarsus yellowish-brown; dorsal surface of mesotibia with erect setae, some plumose, relatively long (1.5 and 1 DE, respectively), plumose setae with very long rachis and branches scarce, restricted to apical third of rachis; mesobasitarsus with a broad band of erect setae and plumose setae, relatively long (1.5 and 1 DE, respectively) and thin setae forming a prominent band in posterior half; plumose setae of posterior edge of metatibia light brown (3 DE), interspersed with longer, thicker, and fuscous setae (4 DE). First metasomal tergum glabrous; TII with very narrow band of tiny bristles along posterior edge, such bristles increasing in length and thickness on succeeding terga, as well as in density and width of band; T5 with longer setae and wider band range, especially medially, but without plumose setae (band of T3 = one-half that of T4; T4 = one-half that of T5); setae of T6 longer and denser (2 DE), intermingled with very thin plumose setae.

#### Metrics.

Total length 4.75; forewing length 5.54; head width 1.99; clypeal width 1.0; clypeal length 0.46; malar length 0.07; compound eye length 1.21; compound eye width 0.55; upper interorbital distance 1.21; maximum interorbital distance 1.26; lower interorbital distance 0.99; alveolorbital distance 0.34; interalveolar distance 0.12; ocellorbital distance 0.30; interocellar distance 0.12; scape length 0.82; scape diameter 0.12; mesofemoral length 1.46; mesofemoral width 0.29; mesotibial length 1.51; mesotibial width 0.34; mesobasitarsal length 0.84; mesobasitarsal width 0.24; metafemoral length 1.88; metafemoral width 0.27; metatibial length 2.76; metatibial width 0.76; metabasitarsal length 0.82; metabasitarsal width 0.37; maximum width of metasomal tergum II 1.34.

#### Distribution.

BRAZIL: States of Ceará (Choró, Maranguape), Rio Grande do Norte (Martins, Mossoró, Natal, Ipanguaçu), Paraíba (Juazeirinho, Santa Luzia), Pernambuco (Cabo de Santo Agostinho, Igarassu), Bahia (Camamu, Catu, Iaçu, Igrapiúna, Itabuna, Itaparica, Lençóis, Maracás, Milagres, Mucugê), and Espírito Santo (Fundão, Guarapari, Jacaraípe, Nova Almeida, Santa Teresa, São Roque).

#### Comments.

There are specimens of *Frieseomelitta meadewaldoi* from Maracás (Bahia, Brazil) labeled by Moure as “*Frieseomelitta luteola* sp. n.” (MS name, *nomen nudum*) in DZUP and it is probable that there are specimens similarly labeled in other collections.

It is of historical interest to note the influence of Brazilian Emperor D. Pedro II who worked tirelessly to bring Brazil to international attention, particularly his endorsement of participation in the Third Universal Exposition of London in 1862 which brought the material studied by Smith (1863). It was at this exposition that various products of Brazil were exhibited, including coffee, mate, rubber, wood, precious stones, machinery, and, of course, bees and their wax and honey, selected from different provinces of Brazil (Almeida 2000). The bees had only vernacular names associated with them and so Smith (1863) was unable to give more precise locality information, simply citing them all as “Brazil”, but he did list these vernacular names (most of Tupi origin) and attempted, where possible, to use them as specific epithets (Smith 1863). According to Camargo and Moure (1996: 110) it is possible that the material was collected in southeastern Brazil, perhaps even the eastern region of the State of Minas Gerais, as evidenced by the etymology of the vernacular names employed.

## Supplementary Material

XML Treatment for Frieseomelitta meadewaldoi
